# Males and females exhibit distinct relationships between intervertebral disc degeneration and pain in a rat model

**DOI:** 10.1038/s41598-020-72081-9

**Published:** 2020-09-15

**Authors:** Grace E. Mosley, Minghui Wang, Philip Nasser, Alon Lai, Daniel A. Charen, Bin Zhang, James C. Iatridis

**Affiliations:** 1 Leni and Peter W. May Department of Orthopaedics, Icahn School of Medicine at Mount Sinai, 1 Gustave Levy, Place, Box 1188, New York, NY 10029-6574 USA; 2 Department of Genetics and Genomic Sciences, Icahn School of Medicine at Mount Sinai, New York, NY USA; 3 Mount Sinai Center for Transformative Disease Modeling, Icahn School of Medicine at Mount Sinai, New York, NY USA; 4 Icahn Institute for Data Science and Genomic Technology, Icahn School of Medicine at Mount Sinai, New York, NY USA; 5 Medical Scientist Training Program, Icahn School of Medicine at Mount Sinai, New York, NY USA

**Keywords:** Peripheral nervous system, Chronic pain, Gene expression, Musculoskeletal system

## Abstract

Back pain is linked to intervertebral disc (IVD) degeneration, but clinical studies show the relationship is complex. This study assessed whether males and females have distinct relationships between IVD degeneration and pain using an in vivo rat model. Forty-eight male and female Sprague–Dawley rats had lumbar IVD puncture or sham surgery. Six weeks after surgery, IVDs were evaluated by radiologic IVD height, histological grading, and biomechanical testing. Pain was assessed by von Frey assay and dorsal root ganglia (DRG) expression of *Calca* and *Tac1* genes. Network analysis visualized which measures of IVD degeneration most related to pain by sex. In both females and males, annular puncture induced structural IVD degeneration, but functional biomechanical properties were similar to sham. Females and males had distinct differences in mechanical allodynia and DRG gene expression, even though sex differences in IVD measurements were limited. Network analysis also differed by sex, with more associations between annular puncture injury and pain in the male network. Sex differences exist in the interactions between IVD degeneration and pain. Limited correlation between measures of pain and IVD degeneration highlights the need to evaluate pain or nociception in IVD degeneration models to better understand nervous system involvement in discogenic pain.

## Introduction

Both low back pain and intervertebral disc (IVD) degeneration are extremely common medical problems^1–3^. IVD degeneration is highly associated with low back pain, and is a common diagnosis in chronic back pain patients^4–9^. The significant global disease burden of chronic low back pain and its strong association with IVD degeneration have resulted in many studies of human patients to identify the associations between degenerative changes seen on spinal imaging and pain presence^5,7,8,10^.

However, the relationship between IVD degeneration and pain is complicated, as nearly one third of asymptomatic controls have been shown to have IVD degeneration in the absence of pain, and imaging findings are not predictive of either development or duration of low back pain^10–12^. Additionally, while associations between spinal measurements and pain have been assessed extensively in human populations, the majority of preclinical research focuses on either IVD degeneration or pain, as recently reviewed^13^.

Few studies have examined the relationships between multiple measurements of IVD degeneration and measurements of nociception or pain^14–18^. Correlational studies within a well-controlled animal model are a priority since they enable measurement of associations between IVD degeneration and pain without the effects of age, and size of the study population that can influence the strength of associations in research using a clinical population. In particular, rat models are ideal for modeling spinal pathology, as rat spines have less spinal phenotypic variation than mouse spines^19^.

It is well known that pain transduction^20–23^ and the development of chronic pain^24–27^ differ between males and females. Female rodents exhibit increased sensitivity to nerve root injury, suggesting sex differences may be present in an IVD degeneration-related (or discogenic) pain model^23^. In humans, the prevalence of low back pain is greater in women than men^28–31^, however, the influence of gender confounds makes it difficult to determine what contribution is due to sex effects. We recently identified sex differences in IVD degenerative changes and fibrotic healing in rats after annular injury^32^. Thus, subtle sex differences in the spine likely interact with nervous system differences. There remains a need for identifying possible sex effects on the interaction between measurements of IVD degeneration and nociception.

Network analysis, long applied to gene expression data sets, has more recently been used to cluster sets of diverse data types^33^. In this type of analysis, correlations between individual variables are presented in an unstructured two-dimensional space to visualize the complex correlative relationships between variables. This network approach allows determination of the specific structural and functional spine measurements most related to distinct quantifications of pain in our model, and how multiple distinct variables may cluster together.

This study determined which measures of structural and functional IVD degeneration are most related to pain measurements in a rat model of low back pain and determined how these relationships between IVD changes and pain assays differ between males and females. A previously developed surgical rat discogenic pain model was used^32,34,35^. We hypothesized that males and females would have distinct correlations within their networks, with more correlations between measures of IVD degeneration and pain found in the male network and estrus stage as a possible confound in the female network.

## Results

### Annular puncture induced structural degenerative changes in lumbar IVDs

**Figure 1:**
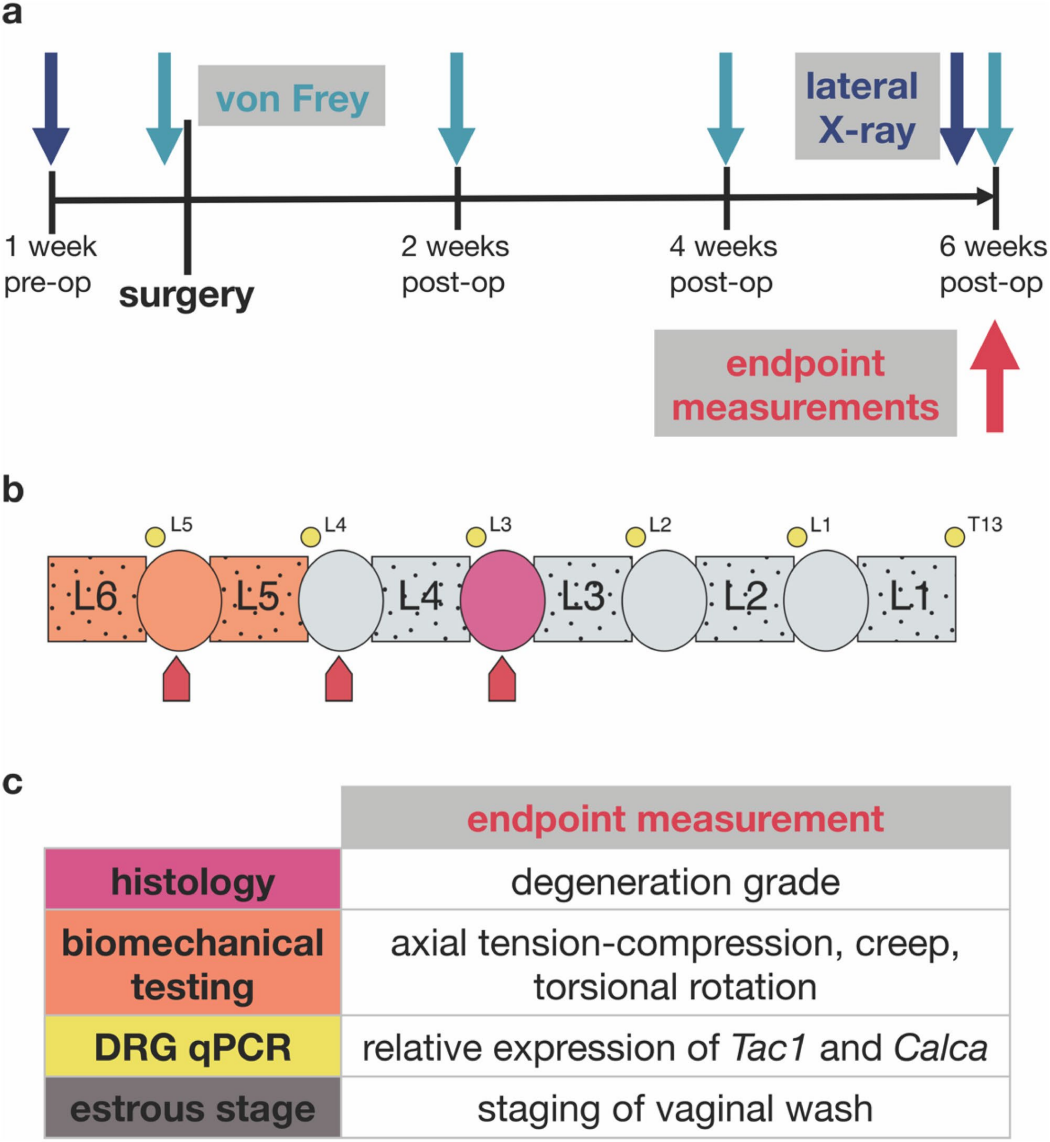
Study design. (**a**) Timeline of X-ray, von Frey, and endpoint measurements. Rats were X-rayed at 1 and 6 weeks, and von Frey testing was performed at weeks 0, 2, 4, and 6. Baseline von Frey was performed 1 day prior to surgery. (**b**) Schematic of sample allocation. Squares represent vertebrae, ellipses IVDs, and small circles DRGs. L3/4, L4/5, and L5/6 IVDs were punctured (red arrowheads). L3/4 IVDs were used for histology (pink), L5/6 motion segments for biomechanical testing (orange), and qPCR was performed on DRGs from the T13–L5 levels (yellow). (**c**) Endpoint measurements included histological degeneration grade, axial tension–compression, creep, torsional rotation, relative DRG expression of *Tac1* and *Calca*, and estrous staging.

We used an annular puncture rodent model of discogenic back pain in order to assess relationships between IVD degeneration and pain. Rats underwent biweekly behavioral pain testing, and several endpoint measures of both IVD degeneration and pain were measured 6 weeks after surgery (Fig. 1).

**Figure 2:**
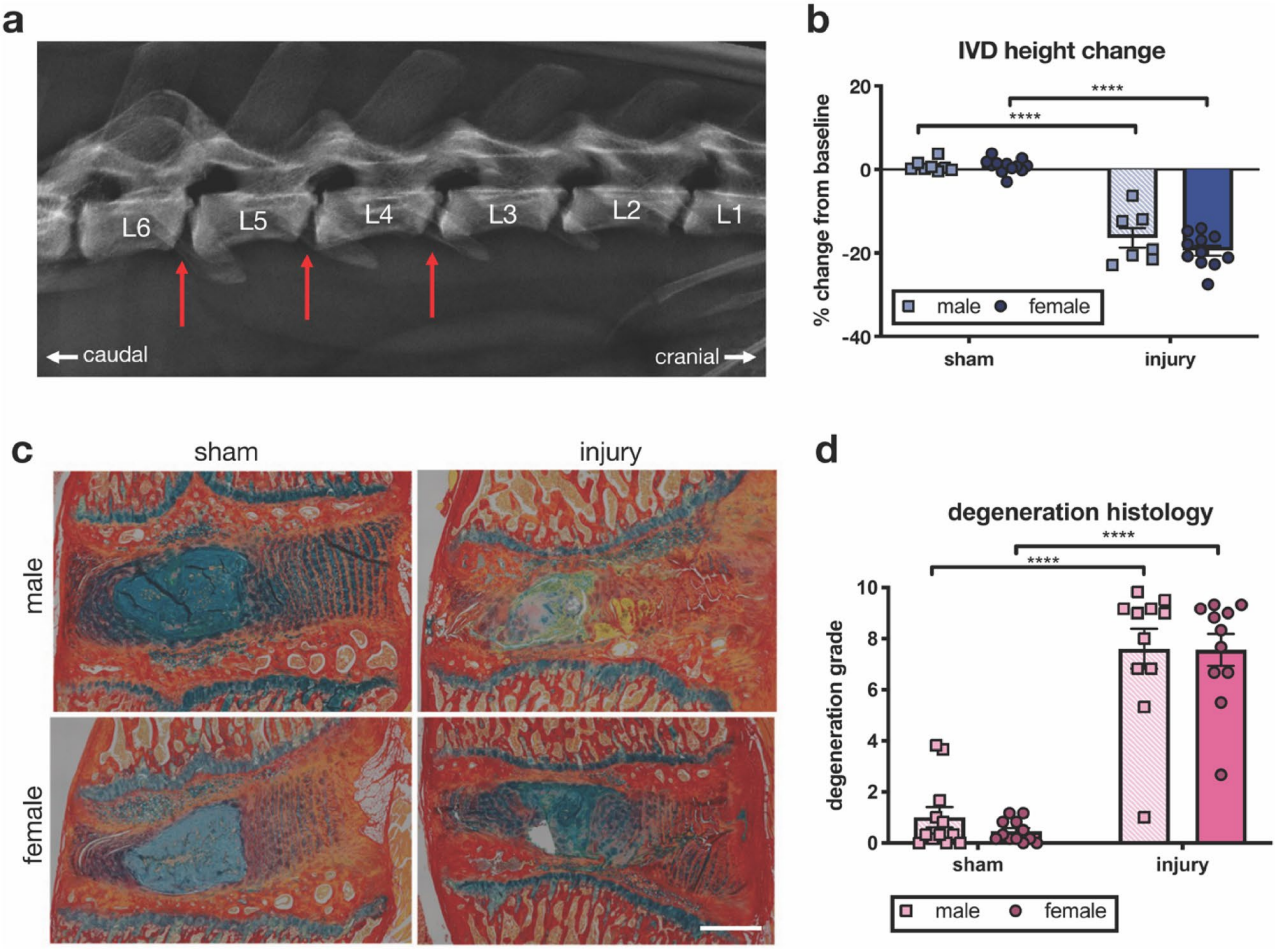
Annular puncture injury induced structural degenerative changes in IVDs. (**a**) Representative lateral radiograph. Red arrows mark L5/6, L4/5, and L3/4 IVDs, which were averaged to measure IVD height. (**b**) Annular injury induced a significant loss of IVD height in both female and male IVDs, as measured by percent change from pre-operative baseline at 6 weeks after injury. (**c**) Representative picrosirius red and alcian blue stained mid-sagittal sections from L3/4 IVDs, with annular disruption and compaction of the nucleus pulposus seen in both females and males. Scale bar = 500 μm. (**d**) Annular injury significantly increased degeneration grade in both female and male IVDs. Degeneration grade was quantified using a semi-quantitative scale evaluating AF integrity, AF/NP border, NP cellularity, NP matrix, and endplate quality (n = 7–12, ****p < 0.0001).

Annular puncture of lumbar IVDs induced macro-structural changes that could be seen via X-ray by measuring IVD height change (Fig. 2a). Baseline size differences in IVD height were accounted for by averaging IVD height of L3/4, L4/5, and L5/6 IVDs (Fig. 2a—red arrows) both at baseline and 6 weeks after injury, and baseline sex differences in IVD height accounted for by reporting IVD height as a percent change from pre-operative baseline. Similar to degenerative grading, change in IVD height showed a highly significant injury effect, but no sex difference (Fig. 2b). When IVD levels were analyzed independently, significant reduction in IVD height was seen in all three punctured levels, but not in adjacent and non-adjacent control IVD levels (see Supplemental Figure S1 online).

In addition to changes in radiologic IVD height, puncture of lumbar IVDs induced histological changes that could be seen 6 weeks after injury. Midsagittal sections stained with picrosirius red and alcian blue exhibited clear disruption of annular lamellae, condensation of the nucleus pulposus, and obscuring of borders between structures (Fig. 2c). These changes after injury were severe, and did not qualitatively differ between females and males, indicating our injury surgery was sufficient to induce lasting changes in collagen organization in females and males. Degeneration grade, determined by a 5-category, 10-point semi-quantitative grading scale, showed a highly significant injury effect, but no effect of sex, affirming qualitative observations (Fig. 2d). Sub-categories in the degeneration scale followed the same pattern as total degenerative grade, with the increase in grade not skewed by one sub-category (see Supplemental Figure S2 online).

### Annular puncture did not cause functional degenerative changes to lumbar IVDs

**Figure 3:**
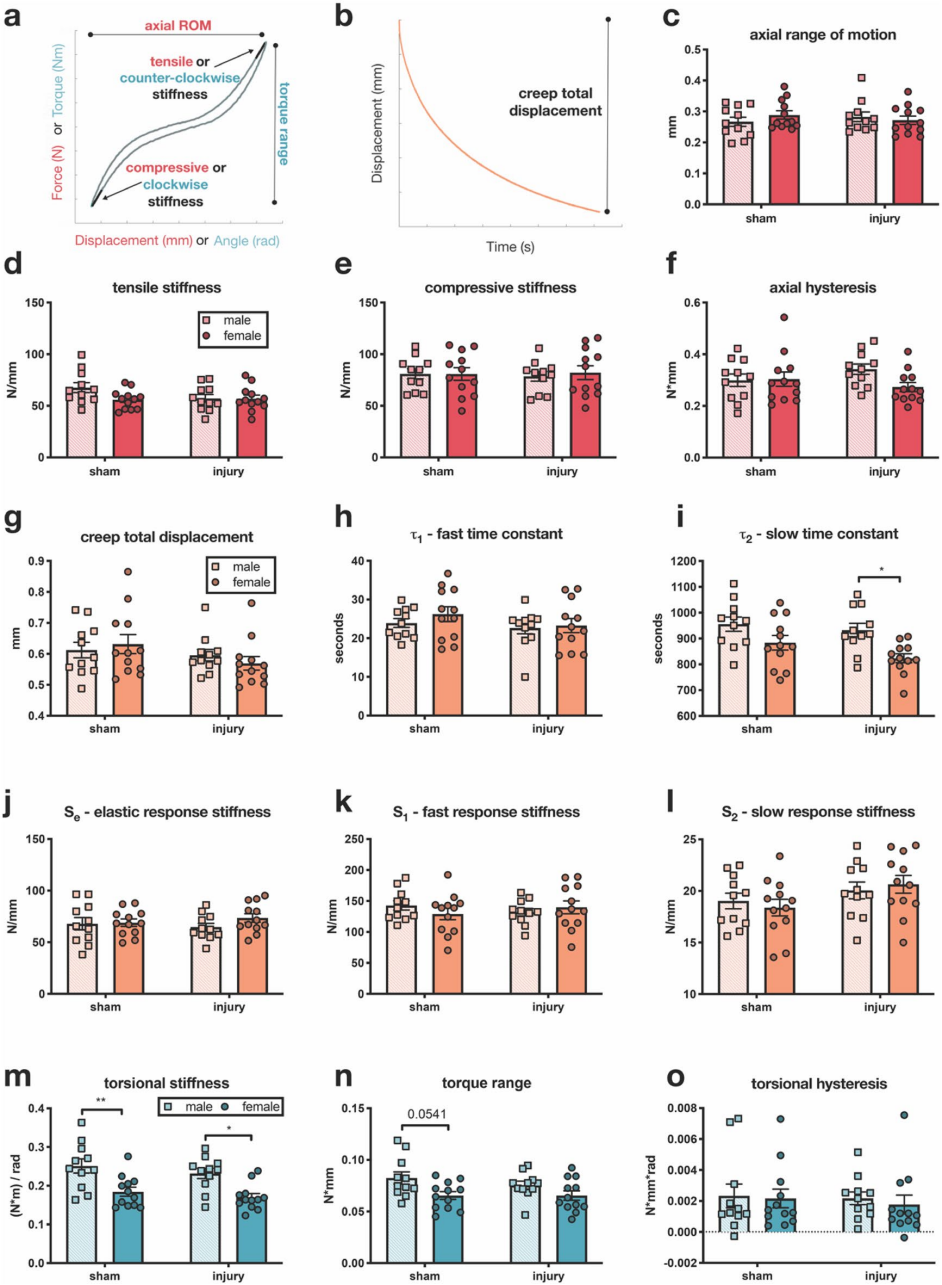
Annular injury resulted in limited functional degenerative changes in IVDs, but some sex differences were observed. (**a**) Representative curve for axial and torsional data. Red text denotes axial components, and blue text denotes torsional components. (**b**) Representative curve for compressive creep data. (**c**–**f**) Axial biomechanical measures showed no injury effect or sex differences at 6 weeks after injury. (**g**–**l**) Creep parameters did not exhibit large changes with injury. Slow response stiffness (**l**) had an injury effect, but it did not persist after post-hoc testing. Males exhibited a greater creep slow time constant (panel **i**) after injury than females. (**m**–**o**) Torsional biomechanical measures showed no injury effect, but sex differences were seen in both torsional stiffness (**m**) and torque range (**n**), with males having greater values. (n = 11–12, *p < 0.05, **p < 0.01).

At 6 weeks after injury, IVD functional changes, as measured by biomechanical testing, did not differ between sham and injured IVDs. Axial and torsional measurements were taken from the loading curve of the last cycle (Fig. 3a) and creep measured after one hour by applying a 5-parameter solid model to the displacement vs. time curve (Fig. 3b). Axial biomechanical testing, measuring compressive and tensile stiffness, axial range of motion, and axial hysteresis, showed neither an injury effect nor a sex effect (Fig. 3c–f). Viscoelastic creep testing, consisting of total displacement, fast and slow exponential time constants, and fast, slow, and elastic response stiffnesses, showed little difference between sham and injury groups (Fig. 3g–l). Slow response stiffness, the stiffness value for the phase of creep that is largely characterized by loss of water content, had an injury effect on 2-way ANOVA, but it was not significant after post-hoc testing (Fig. 3l). Unlike axial testing, a sex difference was seen in creep testing, as males had a greater slow time constant than females after injury (Fig. 3i). The slow time constant is the time constant for the same phase as the slow response stiffness. Torsional biomechanical testing did not show an effect of injury but did exhibit sex effects (Fig. 3m–o). Both torsional stiffness and torque range had a sex effect on 2-way ANOVA, with greater values for males on both tests (Fig. 3m,n).

### Pain following annular puncture showed pronounced sex differences

**Figure 4:**
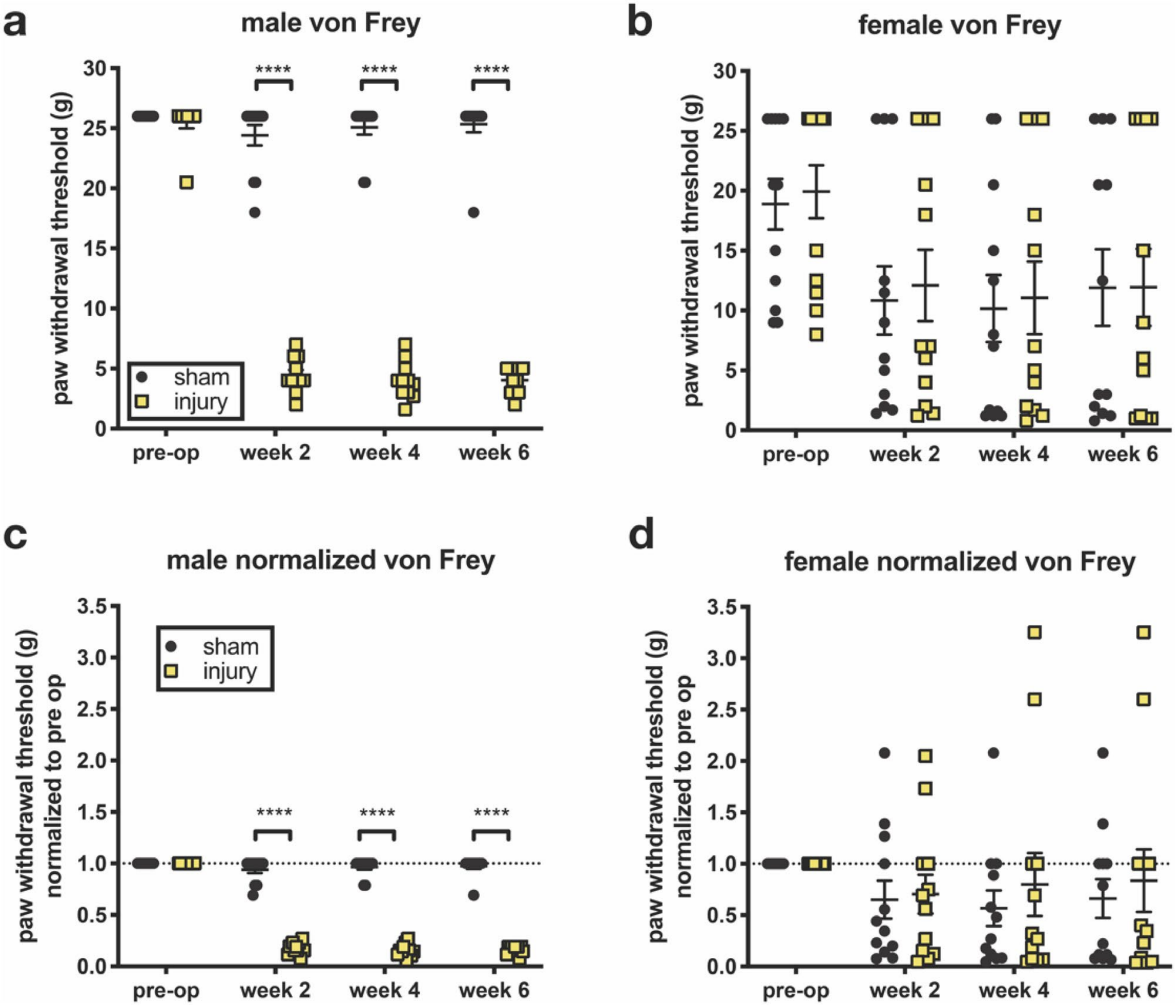
Mechanical allodynia exhibited a pronounced sex effect. Mechanical allodynia was measured using manual application of von Frey filaments. (**a**) Male rats showed a large reduction in paw withdrawal threshold (increase in mechanical allodynia) after injury, which persisted over time. (**b**) Female rats had highly variable paw withdrawal threshold values, and no difference was seen between injury and sham control. (**c**,**d**) Post-operative variability could not be explained baseline variability, as normalizing to baseline did not affect variability of later measurements. (n = 11–12, ****p < 0.0001).

A strong sex effect was seen on mechanical allodynia after IVD injury as measured by the von Frey assay. In male rats, a large reduction in the paw withdrawal threshold was seen at 2, 4, and 6 weeks after annular puncture injury, with no significant change in threshold for sham animals (Fig. 4a). However, in female rats, paw withdrawal thresholds were more variable both at baseline, and after surgery, where paw withdrawal thresholds did not significantly differ between sham and injury groups (Fig. 4b). The variability in the female cohorts could not be explained by the baseline variability, as normalization to pre-operative withdrawal threshold showed a similar pattern as raw values for both sexes (Fig. 4c,d). The female variability was also not due to estrous cycling, as no correlation was found between estrous stage and mechanical allodynia (see Supplemental Figure S3 online).

**Figure 5:**
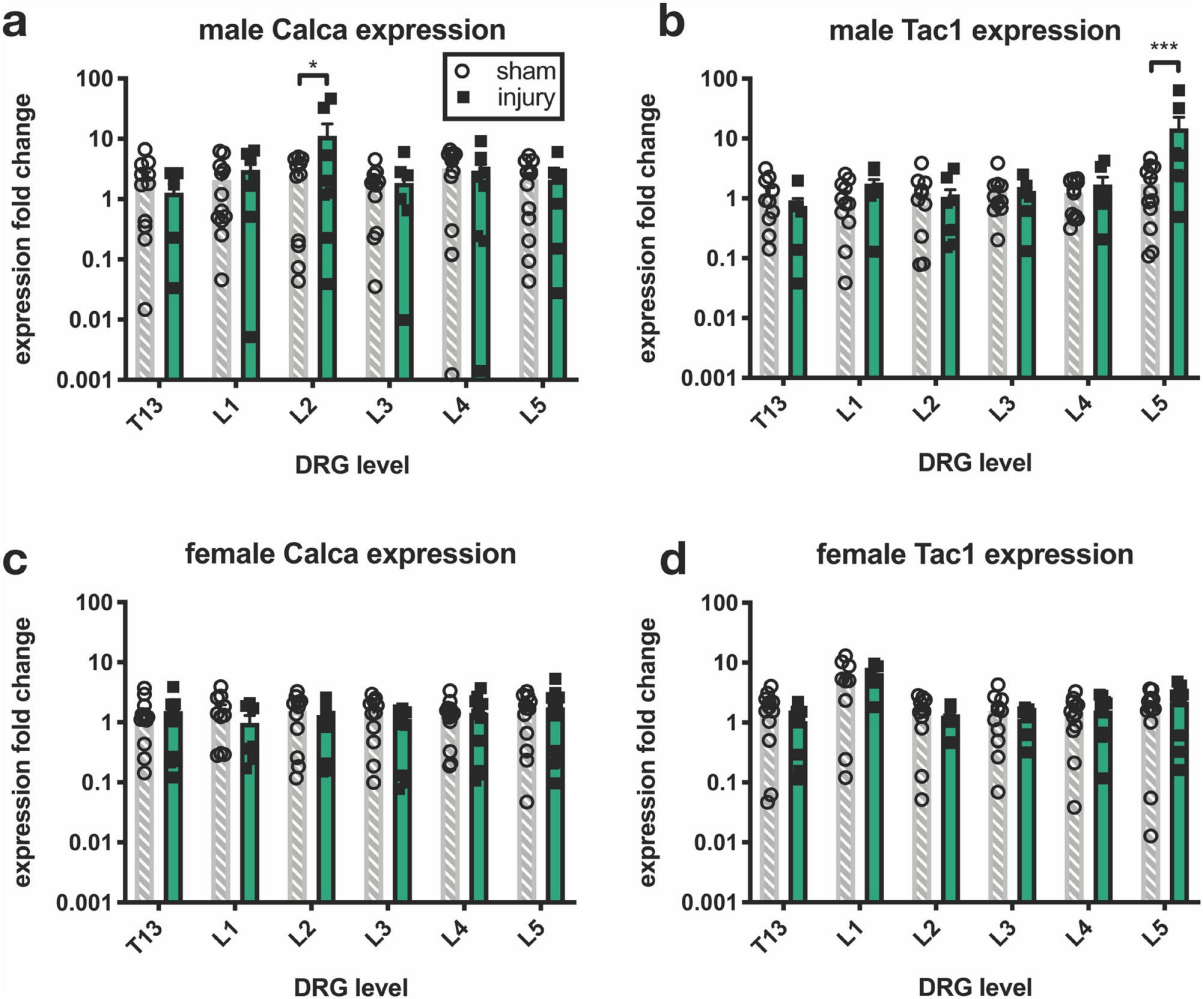
Expression of pain-related genes in lumbar DRGs 6 weeks after injury only differed from sham in male rats, and level effects were observed. (**a**) An increase in *Calca* (coding for CGRP) was observed only in the L2 DRG. (**b**) An increase in *Tac1* (coding for substance P) was only observed in the L5 DRG. (**c**,**d**) No expression changes for *Calca* or *Tac1* were observed in female DRGs. Expression levels were measured relative to expression of the housekeeping gene *Gapdh*, and statistical outliers (as calculated by 1% ROUT in GraphPad Prism) were removed from analysis (n = 11–12, *p < 0.05, ***p < 0.001).

As mechanical allodynia is only one sensory modality of pain, gene expression of known pain-related genes at 6 weeks after injury was measured in the dorsal root ganglia (DRGs), over six spinal levels. An increase in expression of *Calca* (coding for CGRP) and *Tac1* (coding for substance P) was seen after injury only in males (Fig. 5a,b). *Calca* expression was increased after injury at only the L2 level (Fig. 5a). *Tac1* expression was increased after injury at only the L5 level (Fig. 5b). In females, expression of *Calca* and *Tac1* did not differ between sham and injury groups at any DRG level (Fig. 5c,d).

### Correlation networks of IVD and pain measurements differed by sex

**Figure 6:**
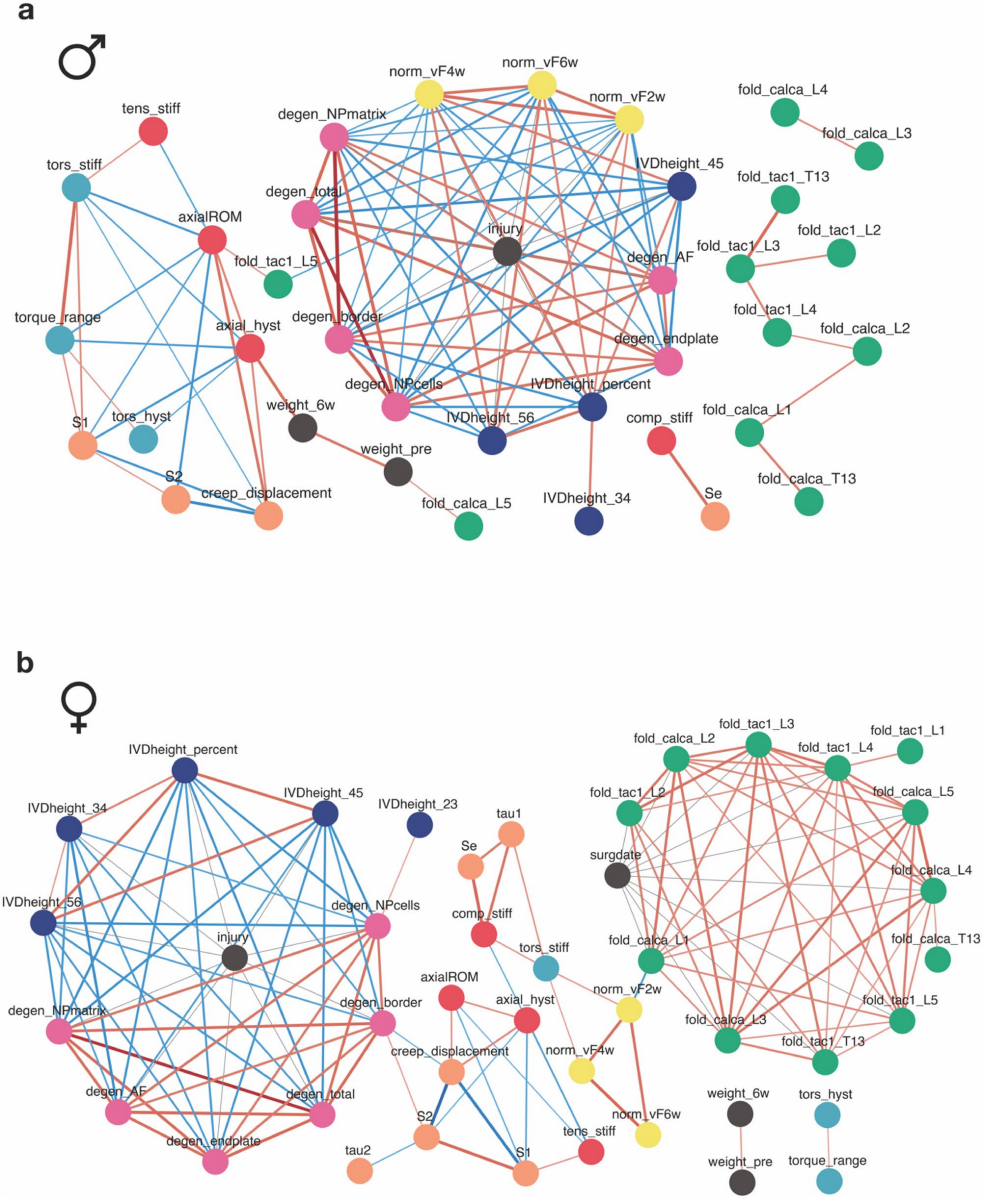
Female and male correlational networks between study variables. (**a**) Male network. (**b**) Female network. Shown are significant correlation pairs at Benjamini–Hochberg’s false discovery rate (FDR) adjusted Spearman correlation p-value ≤ 0.05. Edge color denotes correlation direction (red, positive; blue, negative). Node color denotes variable type.

Pronounced structural differences between male and female correlation networks were observed. In the male network, injury was correlated with radiologic IVD height, histology grading, and von Frey thresholds (Fig. 6a). Interestingly, the change in radiologic IVD height was only significantly associated with injury at the L4/5 and L5/6 levels, not L3/4 or the two adjacent control levels (L1/2 and L2/3) (Fig. 6a), suggesting that IVD height loss after puncture may be larger in more caudal IVDs. As the change in L3/4 height was still correlated with the overall percent change at injured levels, its lack of association with injury is likely the result of a smaller magnitude of IVD height loss, rather than the absence of height loss. Biomechanical tissue properties and DRG pain gene expression were mostly independent of the rest of the male correlation network, and each formed separate clusters (Fig. 6a). While biomechanical measurements were correlated with each other, they were correlated with only a few other study variables, suggesting that at the 6-week time point, functional changes in the IVD were unrelated to structural degeneration and pain. Most gene expression measures were correlated with each other but not the other study variables, which is unsurprising, as these fold changes were all approximately 1. Notable exceptions to the lack of correlation are *Calca* and *Tac1* at the L5 DRG: *Calca* expression is correlated with weight, and Tac1 with both axial range of motion and von Frey threshold at week 2. Thus, even though *Tac1* expression at L5 was not directly correlated with injury, it still appears related to pain phenotype. Details of the correlations between the variables in the males can be found in Supplemental Table S1 online.

In the female network, there was a similar association between injury and radiologic IVD height and histology grading. But unlike the male network, injury was not correlated with any von Frey measurements (Fig. 6b). Further, the change in radiologic IVD height occurred at all three injured levels, as well as the average percent change for injured levels. Additionally, while von Frey measurements were correlated with each other over time and pain qPCR fold changes were correlated across DRG levels, they showed limited correlations with other study variables (Fig. 6b). However, similar to the link between early von Frey threshold and *Tac1* expression in the male network, in the female network, the von Frey threshold at week 2 was correlated with expression of *Calca* in the L1 DRG, suggesting that it may still be important for the pain phenotype, even though its expression was not significantly correlated with injury group. Interestingly, a link between von Frey and torsional stiffness was also present in the female network. Thus, torsional stiffness may be an important functional measurement of the IVD for the development of back pain in females. In humans, torsional rotation differs between healthy and unhealthy IVDs, and further differs with the presence of pain, supporting torsional biomechanics as particularly relevant for back pain^36^. This also suggests that there may be distinct relationships between integrity of the outer AF and pain in males and females, as torsional stiffness is predominantly influenced by the integrity of the outer AF^37^. Similar to the male network, biomechanical properties showed few correlations with other variable types. Still, there were some connections between creep parameters and the quality of the AF/NP border, and between torsional stiffness von Frey thresholds. Finally, DRG gene expression measurements were strongly correlated with each other, but not with the other study variables. The amount of intercorrelation in this cluster is unsurprising, given most fold changes were close to 1. A correlation with date of surgery was detected, suggesting there may be slight variation cohort to cohort. Importantly, estrous cycle stage was not significantly correlated with any study variables, demonstrating that variability in some female measurements, as well as sex differences between the male and female networks, are unlikely to be due to changes in hormonal levels in the females. Details of the correlations between the variables in the females can be found in Supplemental Table S2 online.

## Discussion

This study identified relationships between structural and functional measures of IVD degeneration and measures of pain by using network analysis to construct sex-specific correlation networks. We hypothesized that females and males would differ in their relationships between IVD injury and pain with more correlation between measures of pain and IVD degeneration in the male network, due to previous findings of subtle sex differences in fibrotic IVD degenerative changes^32^ and known sex differences in pain^20,21^. The most important findings were the sex differences between the correlational networks where injury was associated with pain measures in males and not in females. Radiologic IVD height measurements and histological grading correlated with injury group in both females and males, indicating that IVD degeneration occurred regardless of sex. Contrary to our initial hypothesis, the estrus cycle did not correlate with any variables in the female network.

In low back pain patient populations, pain is often not correlated with imaging findings and is poorly indicated for surgical repair^10,11,38–40^. This rat model study assesses how a variety of measurements of IVD degeneration relate to measures of pain, in order to both understand how our model relates to the human condition, and to determine which measures of IVD degeneration appear most relevant for the development of pain. Taking both female and male rat cohorts together, our results emulate some of the complexities in the relationship between back pain and IVD degeneration observed in humans and provides some additional evidence for this model’s relevance. In our study, the correlation in males between IVD injury and pain and lack of a significant correlation in females also highlight the importance of sex as a major source of variance in these relationships.

Sex differences have been observed in many other models of pain, including sciatic nerve injury^25^, chronic constriction injury^41,42^, formalin injection^43,44^, and radiculopathy models^23^, as well as within human back pain patient populations^45–47^. In these prior studies, pain in females and males involved different immune cell populations and modulated expression of different genes, and more commonly showed more severe phenotypes in females. While women report more back pain than men^28^, this is influenced by effects of both sex and gender, and a different pattern of pain in animal studies may be due to the absence of gender effects. Sex differences in animal models of low back pain have been underexplored, but recent work from Millecamps and Stone using female mice found a similar lack of difference in mechanical allodynia after IVD puncture injury up to 1 year after injury, as measured by the von Frey assay^48^, similar to the findings of this study. Thus, the lack of correlation between injury and mechanical allodynia may mean that in females, sensitivity to mechanical stimuli is not impacted by IVD injury and degeneration. Additionally, this sex difference cannot be attributed to an effect of estrus stage, as final von Frey threshold did not differ across estrous stages (ANOVA, p = 0.664) (see Supplemental Figure S3 online). Estrus stage has been shown to influence response to pain from electrical stimulation^49^, but as such a stimulus bypasses ion channels on sensory neurons, it may not be relevant to our model.

Given the high variability in female paw withdrawal thresholds for both sham and injured animals both at baseline and following surgery, we believe the sex difference observed may also be influenced by stress. Females are more sensitive to acute stress than males^50–52^, so the acute stress of the testing environment may be sufficient to influence female but not male von Frey responses. In this study, stress on the animals was minimized with acclimation to both handling and testing apparatus, and with our decision to avoid any other evoked pain behavioral measurements, but it remains possible that this acclimation process was insufficient for the females. Stress and pain are known to interact^53,54^, and depression and anxiety have been shown to be highly associated with chronic low back pain in human patient populations^55–65^. Thus, while an experimental confound, stress plays an important role in the presentation of chronic low back pain. Additionally, it is possible that the sham surgery has a larger effect in females, as the injury surgery was developed in male rats^35^. Future work with behavioral assays to more thoroughly characterize affective state is warranted to better understand how stress may influence the sex differences observed in this study.

Pain gene expression varied across DRG levels and exhibited sex differences. Broad investigations of DRG gene expression in radiculopathy models have demonstrated changes after application of IVD tissue to nerve roots^66–68^, and IVD puncture without direct nerve damage has been previously shown to influence gene expression at 1 and 3 days after injury^69^. Substance P and Calcitonin gene-related peptide, encoded by *Tac1* and *Calca*, respectively, are classically involved in pain transduction, and sensory neurons innervating lumbar IVDs have been shown to express both proteins^70^. Substance P protein expression in rat L1 DRGs was significantly increased at 6 weeks following a similar IVD injury^34^. In this study, females showed no significant changes in expression between sham and injury at 6 weeks, but males showed increased expression for *Calca* and *Tac1*, each at a single DRG. While sex differences may be a result of sex-specific molecular pathways for nociception, it is also possible that temporal differences in nociceptive PNS changes between males and females exist, as our gene expression data only reflected the DRG gene expression at 6 weeks after injury.

Level differences in DRG gene expression may suggest two distinct pain pathways. In males, *Calca* and *Tac1* gene expression levels increased following injury at the L2 and L5 DRGs, respectively. The network analysis only detected significant correlations between pain-related gene expression and other variables at the L5 DRG in males and L1 DRG in females. These findings suggest two distinct pain transduction pathways from the lumbar IVDs—one at lower DRG levels adjacent to injured IVDs, and one at higher DRG levels, several levels cranial to the injured IVDs. Similar findings have been observed in retrograde tracing studies examining the innervation of lumbar DRGs. Both dorsal and lateral aspects of the L5/6 IVD in rats are innervated by sensory neurons from L6 to T13 DRGs, with neurons travelling to L3–L6 DRGs via the sinuvertebral nerve, but T13–L2 DRGs through the paravertebral sympathetic trunks^70–72^.

Some limitations of this study warrant discussion. To construct the correlation network, all measurements had to be performed within the same animals. Thus, in an effort to not over-stress the rodents, a single sensory modality was used for behavioral pain measurements. Von Frey was selected over other assays as it was previously shown to be sensitive to IVD injury in rats^35^. It is possible that the sex differences in mechanical allodynia are not present in other pain modalities, such as heat or cold. In anterior IVD puncture models that used behavioral assays for hyperalgesia or allodynia, separate studies found development of hindpaw cold hyperalgesia 12 months after injury^48^ in females, while tail cold allodynia took 14 days to develop in males^15^. Males also did not show hindpaw heat hyperalgesia up to 45 days after injury^16^ or tail heat allodynia up to 14 days after injury^15^. While both species and assay choice differences limit comparisons, there do appear to be temporal differences in pain development between sexes. Molecular measures of nociception prioritized an assessment of differences between spinal DRG levels and looked at classical pain-related genes *Calca* and *Tac1.* It remains possible that other pain-related genes, including, but not limited to ion channels, cytokine receptors, and opioid receptors, may be uniquely affected by IVD injury and degeneration, and larger studies of gene expression changes at both the peripheral and central nervous system level at this chronic time point are needed. The lack of injury effect on biomechanical testing is likely a result of fibrotic healing that functionally restored biomechanical tissue properties. This is supported by Ashinsky et al., who found that IVD biomechanics after injury change over time, and found a similar lack of differences from controls in biomechanical properties at 4 and 8 weeks after injury^73^ due to functional fibrotic healing. Importantly, this work explores correlations and further work is needed to determine the mechanism for the relationships between variables.

In conclusion, this work found distinct responses to IVD injury by sex, by analyzing structural and functional IVD degeneration, as well as mechanical allodynia and DRG gene expression 6 weeks after IVD injury. Correlational network analyses showed that males demonstrated clear relationships between injury, structural IVD degeneration, and mechanical allodynia, while females did not. The limited correlations between spine and pain measures in females may be a result of increased sensitivity to the acute stress of the behavioral tests. This research highlights the need to treat female and male animals as distinct cohorts and suggests a need for future work on low back pain models examining how these spine and nervous system relationships may shift over time and across pain modalities.

## Materials and methods

### Animals

All procedures were reviewed and approved by the Institutional Animal Care and Use Committee at the Icahn School of Medicine at Mount Sinai and are consistent with animal care guidelines. Forty-eight (24 male, 24 female) 4-month old virgin skeletally mature Sprague–Dawley rats (Charles River Laboratory, Wilmington, MA) were used in this study. Rats were divided into four groups: male sham, female sham, male injury, and female injury, with random assignment into sham and injury groups (n = 12 per group). Timeline of study design is depicted in Fig. 1a. Rats underwent either a sham surgical procedure or annular puncture surgery of the L3/4, L4/5, and L5/6 IVDs. Sham surgery consisted of an anterior abdominal incision through both skin and peritoneal membrane, from approximately the xiphoid process to the iliac crest, followed by exposure of anterior surface of L3/4, L4/5, and L5/6 IVDs. Sham animals did not receive any needle puncture in IVDs. IVD level was determined using preoperative anterior X-ray images, and anatomical landmarks (aortic trifurcation visualization and iliac crest palpation). Injury surgery used the same surgical approach as sham, but the exposed IVDs were each punctured three times (midline anteriorly, and left and right anterolaterally) with a 26G needle and the midline puncture included an injection of 2.5μL of 0.1 ng/μL TNF-alpha to induce an inflammatory response^32^. 26G needle is 42–46% and 46–52% of IVD height for males and females, respectively, and significant sex differences in IVD height are only found at the L3/4 level^32^. Such size difference is only 4–6% of the total IVD height, and in both males and females, all punctured levels are injured with needles > 40% of IVD height, which has been demonstrated to be sufficient to induce degenerative IVD changes^74^. All surgical procedures were performed under 2–3% inhaled isoflurane. One animal (male injury) died from surgical complications. Intervertebral disc puncture was confirmed with histology, and one animal (female injury) was excluded due to mispuncture of IVD. Animals maintained a Body Conditioning Score > 2. Animals were allowed unrestricted movement in their home cages for the entire 8 week experimental duration and housed two per cage with the exception of the 24 h post-operative period, when animals were singly housed. Animals were maintained at a 12/12 h light/dark cycle (light stage: 7 am to 7 pm). IVD and DRG levels were uniformly allocated for analysis as depicted in Fig. 1b for endpoint measurements listed in Fig. 1c.

### Estrous staging

Vaginal washes were performed on female rats at time of euthanization. About 150 μL of sterile phosphate buffered saline was pipetted in and out of the vagina, onto a microscope slide, and allowed to dry. Slides were then stained using the Shorr method^75^, imaged at 20 × (Leica Microsystems, Wetzlar, Germany), and classified as estrus, metestrus, diestrus, or proestrus, based on the criteria described by Paccola et al.^76^.

### Histology

L3/4 motion segments (vertebra-IVD-vertebra) were fixed, decalcified, paraffin-embedded and sagittally sectioned at 5 µm, and sections from the midsagittal region were used for degeneration grading (Fig. 1b,c). Sections were stained for collagen and glycosaminoglycans with picrosirius red and alcian blue (PR/AB), respectively. PR/AB stained sections were evaluated by three blinded evaluators using a semi-quantitative system to determine degeneration grade, that evaluates annulus fibrosus (AF) integrity, nucleus pulposus (NP) cellularity, NP matrix quality, interruption of AF/NP border, and endplate irregularity, that is sensitive to puncture-induced degenerative changes^32,77^.

### Radiology

Height of the lumbar IVDs was assessed with in vivo lateral X-rays, both pre-operatively and at 6 weeks post injury. Rats were anesthetized with inhaled isoflurane for 10 min to ensure consistent muscle relaxation and were X-rayed at 55 kV for 10 s (UltraFocus Faxitron, Tucson, AZ). Cranial and caudal vertebral borders were manually defined in Fiji (National Institutes of Health, Bethesda, MD), and IVD height calculated using a MATLAB (MathWorks, Natick, MA) code which has been described previously^32^. The absolute height for individual IVDs was measured and percent change from baseline for the average across L3/4, L4/5, and L5/6 was calculated.

### Biomechanics

Biomechanical testing was performed on the L5/6 motion segments (vertebra-IVD-vertebra) from each animal (Fig. 1b,c). Testing consisted of 20 cycles of axial tension–compression sinusoidal loading at ± 8 N and 1 Hz, followed immediately by 1 h of compressive creep at − 8 N. After 30 min of unconstrained rehydration in saline, 20 cycles of torsional rotation was applied at ± 10° and 1 Hz, with 8 N axial static compression^32^. Axial and creep testing were done using a TA ElectroForce 3200 instrument (TA Instruments, New Castle, DE) and torsional testing with an AR2000ex rheometer (TA Instruments, New Castle, DE). Axial and torsional properties were calculated from the 20th loading cycle using MATLAB^32^ and creep parameters were determined by applying a 5-parameter viscoelastic solid model^32,78^. The viscoelastic model is the sum of a rapid and slow exponential decay and an elastic component.

### Behavioral analysis

Mechanical allodynia was assessed using the von Frey assay^35,53^. Rats were acclimated to handling and test cages for 7 consecutive days prior to testing. Rats were tested pre-operatively and at 2 week intervals thereafter. On testing day, rats were allowed to acclimate to the test cages for 20 min before testing. Male and female rats were tested on separate days. All testing was performed by a single blinded experimenter and took place between the hours of 19:00 and 21:00, during the dark cycle phase. Von Frey filaments ranging in force from 0.4 to 26.0 g were applied in ascending force, with each filament applied five times to the plantar surface of the hind paws. The lowest force filament which elicited nocifensive behaviors in 3 of 5 applications was considered the paw withdrawal threshold, and paw withdrawal threshold reported and the average between the left and right hindpaws. Nocifensive behaviors included paw licking, extended paw withdrawal, and fanning/shaking of the paw.

### qPCR

Dorsal root ganglia (DRG) expression of the pain-related genes *Calca* (calcitonin gene-related peptide, CGRP) and *Tac1* (substance P), known to be important in peripheral nociceptive neurons^79^, was measured using quantitative PCR. A significant percentage of neurons innervating the rat IVD have been demonstrated to be sCGRP+ and/or Substance P+^70,80,81^, and expression of genes encoding for each has been previously shown to be significantly upregulated in DRGs 4–8 weeks after IVD injury in rats^15,82^. Total RNA was extracted from pooled left and right DRGs from a single spinal level using Trizol and purified using the RNeasy Micro Kit with DNase application (Qiagen, Hilden, Germany), and converted to cDNA using the Affinity script Reverse Transcriptase system (Agilent Technology, Santa Clara, CA). Spinal levels from T13 to L5 were independently analyzed (Fig. 1b). qPCR was performed on samples with an OD260/280 value of 1.7 or greater using Taq Polymerase Platinum (Life Technologies, Carlsbad, CA) in an ABI PRISM 7900HT sequence detection systems with robotic arms (Applied Biosystems, Foster City, CA). Expression levels were measured relative to expression of the housekeeping gene *Gapdh*, and statistical outliers (as calculated by 1% ROUT in GraphPad Prism) were removed from analysis. Primer sequences can be found in Supplemental Table S3 online.

### Network analysis

All previously described measurements were performed within single animals, allowing for the construction of trait-trait correlation networks. Spearman’s correlation analysis was calculated using the R programming language to determine significant correlations between measurements with Benjamini–Hochberg’s false discovery rate (FDR) corrected p ≤ 0.05^83^. Male-only and female-only trait correlation networks were constructed to investigate differences in relationships between all 78 variables measured by sex (see Supplemental Table S4 online). While there is a limited power to detect correlations with small or medium effect sizes given the current sample size (24 in each sex group), we expected to achieve a relatively high (~ 73%) power to detect correlations of large effect size at a p value significance level of 0.05 according to Cohen’s guidelines^84^. Network visualization was generated with Cytoscape (v 3.6)^85^. While the majority of measurements included were all taken at the 6 week time point, pre-operative weight was included as a control (to ensure appropriate correlation with 6-week weight), and earlier von Frey time points were included to evaluate whether baseline sensitivity or earlier post-operative mechanical allodynia may be predictive of more chronic IVD and DRG changes.

### Statistical analysis

Statistical analysis for IVD and pain measurements used Prism (GraphPad, La Jolla, CA) using p < 0.05 to identify statistically significant differences. Differences between groups were evaluated with 2-way ANOVA (injury and sex) for radiologic IVD height, histological grade, and biomechanical properties. 2-way ANOVA (injury and time) was used for von Frey thresholds, and 2-way ANOVA (injury and DRG level) was used for pain gene expression measurements, and males and females were analyzed independently. Tukey’s post-hoc test was used for all analyses of variance.

## Supplementary information

Supplementary Information.
