# Supplementary material for: Males and females exhibit distinct relationships between intervertebral disc degeneration and pain in a rat model
Source: Sci Rep. 2020 Sep 15;10:15120. doi: 10.1038/s41598-020-72081-9 (PMC7492468; doi:10.1038/s41598-020-72081-9)
Supplement: Supplementary file 1 — Supplementary Information. [file 41598_2020_72081_MOESM1_ESM.docx]

**Supplemental Figures and Tables**

**
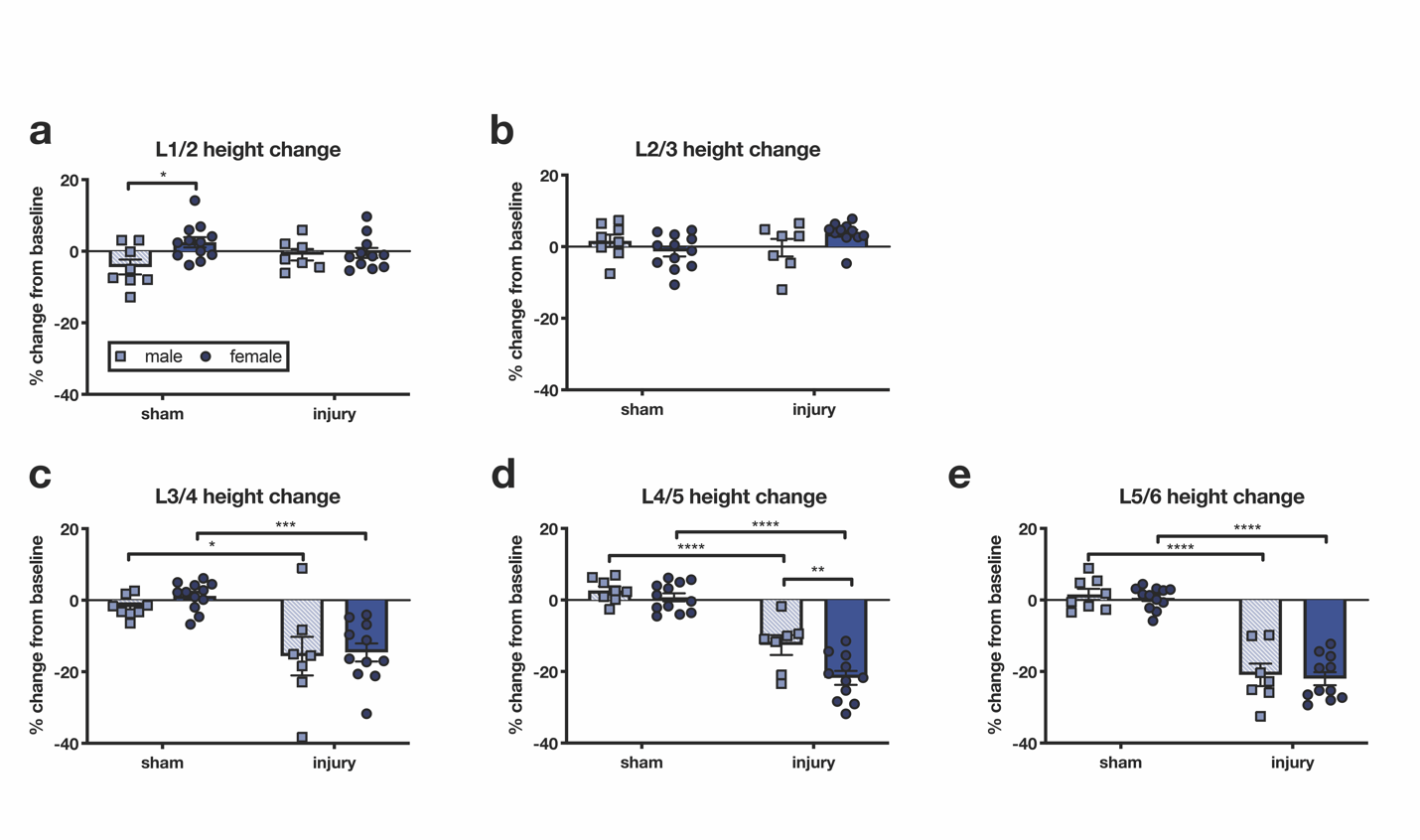
**

**Supplemental Figure S1: Annular puncture injury caused a reduction of IVD height at punctured levels, but not adjacent and non-adjacent unpunctured levels.** (A) No difference in IVD height at a non-adjacent unpunctured IVD was seen between sham and injury for either males (p = 0.5472) or females (p = 0.4772). (B) No difference in IVD height at an adjacent unpunctured IVD was seen between sham and injury for either males (p = 0.8675) or females (p = 0.0680). (C-E) All three punctured IVD levels had a reduction in IVD height at 6 weeks after injury in both males and females. (n = 7-12, * = p<0.05, ** = p<0.01, *** = p<0.001, **** = p<0.0001).

**
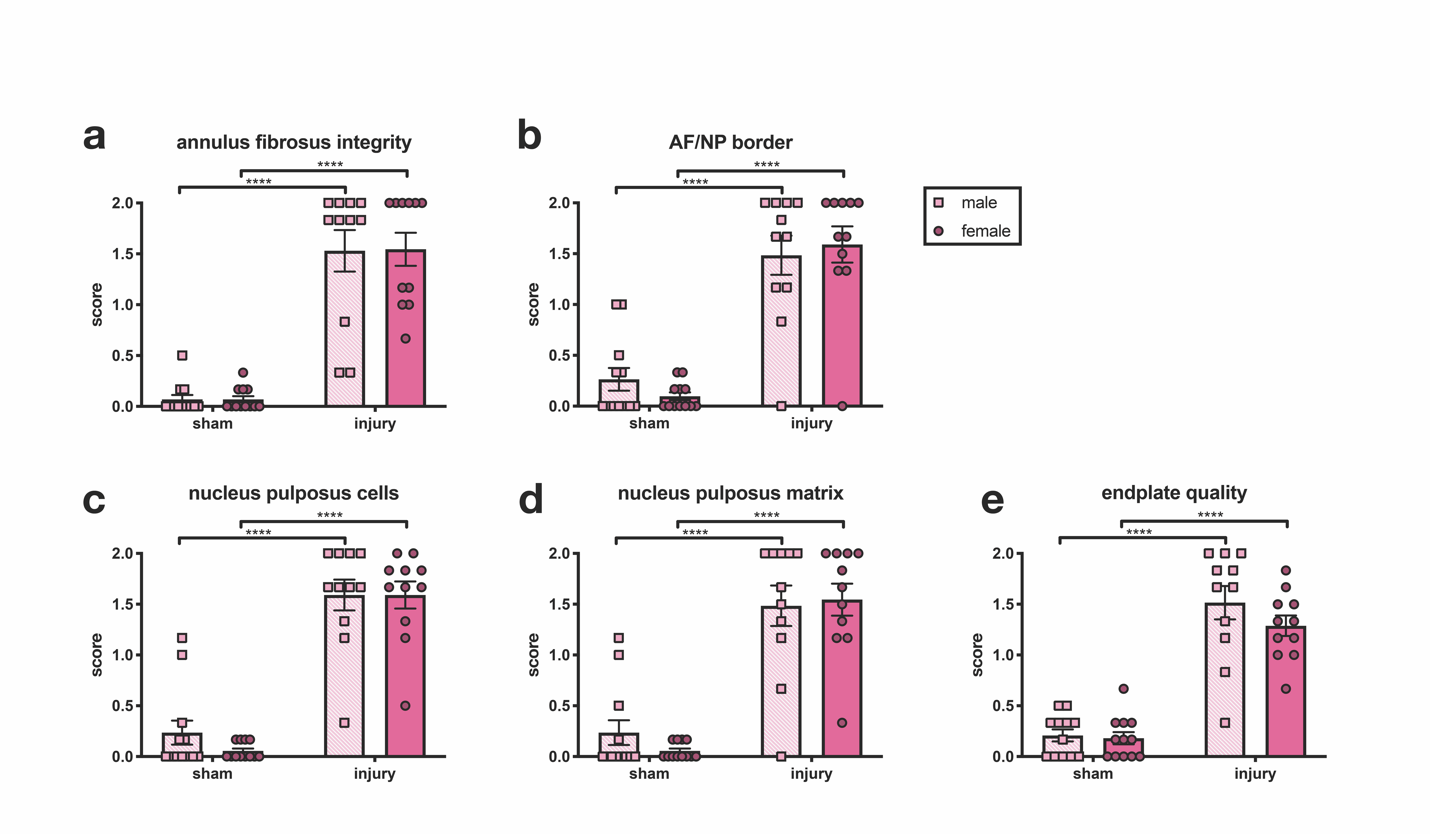
**

**Supplemental Figure S2: Sub-categories of degeneration grading scale showed similar effects as total degeneration grade.** (A-E) All subcategories for degeneration graded showed highly significant injury effects, but no sex differences. (n = 11-12, **** = p<0.0001).

**
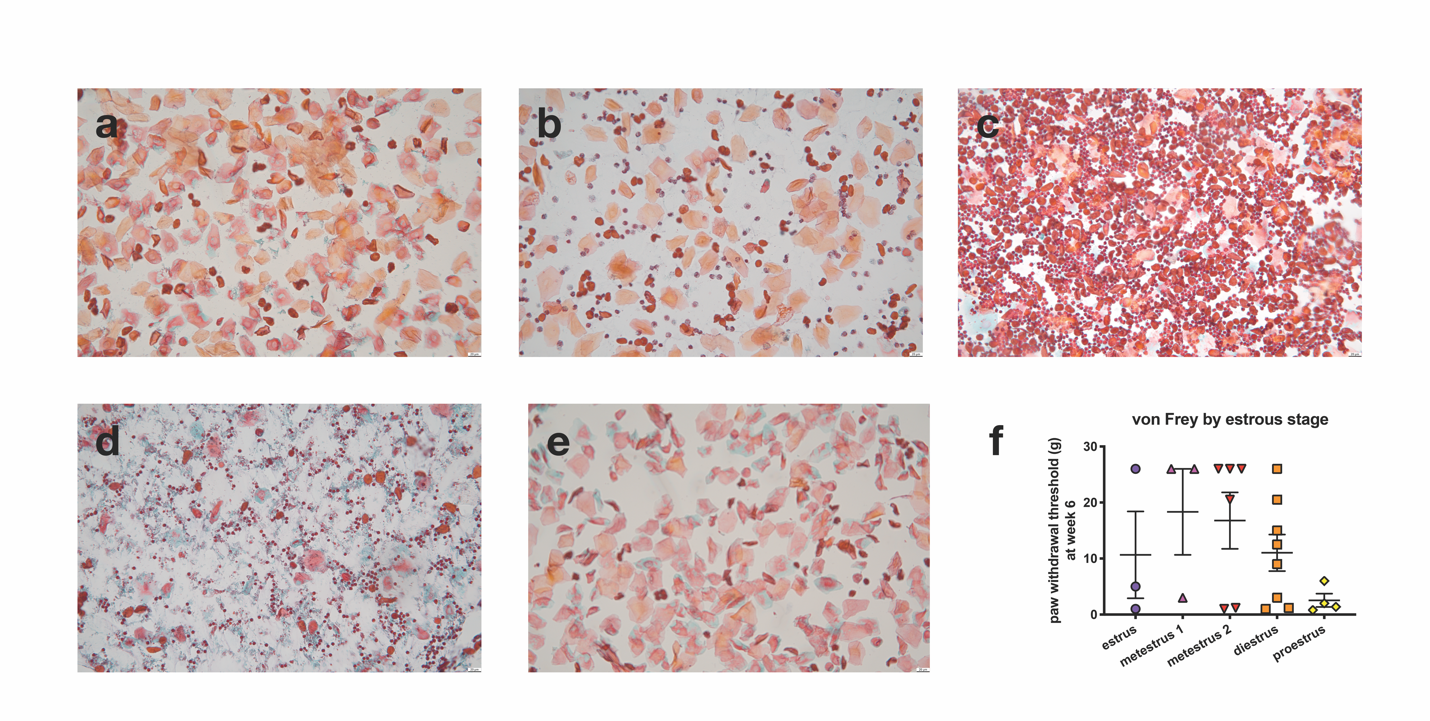
**

**Supplemental Figure S3: Estrous stage did not influence von Frey paw withdrawal threshold.** (A-E) Representative Schorr-stained vaginal smears from estrus (A), metestrus 1 (B), metestrus 2 (C), diestrus (D), and proestrus stages (E), respectively, measured at euthanization (6 weeks post-operative). (F) Paw withdrawal threshold at 6 week timepoint was not significantly correlated with estrous stage (p = 0.2632).

**Supplemental Table S1. Male-specific correlation network analysis.**

| Variable 1 | Variable 2 | Rho | p | p.adj |
| --- | --- | --- | --- | --- |
| injury | norm_vF6w | NA | 3.68E-17 | 3.63E-14 |
| injury | norm_vF4w | NA | 7.60E-17 | 3.75E-14 |
| degen_NPmatrix | degen_border | 0.97748079 | 5.52E-15 | 1.81E-12 |
| injury | norm_vF2w | NA | 1.75E-14 | 4.31E-12 |
| degen_total | degen_NPcells | 0.958656461 | 2.22E-12 | 4.38E-10 |
| degen_total | degen_border | 0.942970445 | 5.18E-11 | 8.53E-09 |
| degen_total | degen_NPmatrix | 0.93327152 | 2.39E-10 | 3.38E-08 |
| torque_range | tors_stiff | 0.928853755 | 4.46E-10 | 5.50E-08 |
| degen_NPmatrix | degen_NPcells | 0.924623335 | 7.81E-10 | 8.56E-08 |
| degen_NPcells | degen_border | 0.922091226 | 1.08E-09 | 1.06E-07 |
| degen_total | degen_endplate | 0.90220285 | 9.60E-09 | 8.61E-07 |
| norm_vF4w | norm_vF2w | 0.897655424 | 1.48E-08 | 1.22E-06 |
| degen_total | degen_AF | 0.893850978 | 2.10E-08 | 1.60E-06 |
| Se | comp_stiff | 0.890708875 | 2.78E-08 | 1.96E-06 |
| norm_vF6w | norm_vF4w | 0.877903301 | 7.95E-08 | 5.05E-06 |
| degen_NPcells | degen_AF | 0.87753087 | 8.19E-08 | 5.05E-06 |
| injury | degen_endplate | NA | 3.15E-07 | 1.78E-05 |
| norm_vF6w | norm_vF2w | 0.857995742 | 3.30E-07 | 1.78E-05 |
| fold_tac1_L3 | fold_tac1_T13 | 0.911764706 | 3.51E-07 | 1.78E-05 |
| degen_endplate | degen_NPcells | 0.856702873 | 3.60E-07 | 1.78E-05 |
| creep_displacement | S2 | -0.855448899 | 3.90E-07 | 1.83E-05 |
| injury | degen_total | NA | 4.49E-07 | 2.01E-05 |
| creep_displacement | axialROM | 0.849802372 | 5.59E-07 | 2.40E-05 |
| injury | degen_AF | NA | 9.90E-07 | 4.07E-05 |
| degen_endplate | degen_AF | 0.833652224 | 1.45E-06 | 5.71E-05 |
| injury | degen_NPcells | NA | 1.51E-06 | 5.73E-05 |
| degen_border | degen_AF | 0.806938607 | 5.70E-06 | 0.000208186 |
| injury | IVDheight_percent | NA | 6.17E-06 | 0.000217601 |
| degen_endplate | degen_border | 0.802365994 | 7.05E-06 | 0.000240011 |
| degen_NPmatrix | degen_AF | 0.794356085 | 1.01E-05 | 0.000333047 |
| degen_endplate | degen_NPmatrix | 0.786922106 | 1.40E-05 | 0.000444644 |
| norm_vF6w | degen_NPcells | -0.777723632 | 2.04E-05 | 0.000630653 |
| creep_displacement | S1 | -0.765104461 | 3.35E-05 | 0.000974697 |
| norm_vF6w | degen_total | -0.764739052 | 3.40E-05 | 0.000974697 |
| norm_vF6w | degen_AF | -0.764231566 | 3.47E-05 | 0.000974697 |
| injury | degen_border | NA | 3.58E-05 | 0.000974697 |
| S2 | axialROM | -0.76284585 | 3.65E-05 | 0.000974697 |
| axial_hyst | weight_6w | 0.876923077 | 3.83E-05 | 0.000994714 |
| injury | degen_NPmatrix | NA | 4.97E-05 | 0.001257702 |
| S1 | axial_hyst | -0.751552795 | 5.53E-05 | 0.001363686 |
| tors_stiff | axialROM | -0.744776962 | 7.01E-05 | 0.001688085 |
| norm_vF2w | degen_endplate | -0.744024611 | 7.20E-05 | 0.001691281 |
| IVDheight_percent | IVDheight_56 | 0.859340659 | 8.20E-05 | 0.001882414 |
| axial_hyst | axialROM | 0.739130435 | 8.50E-05 | 0.001907732 |
| injury | IVDheight_56 | NA | 0.000102365 | 0.002245204 |
| norm_vF6w | degen_endplate | -0.731379137 | 0.000109985 | 0.002359897 |
| creep_displacement | axial_hyst | 0.72783738 | 0.000123347 | 0.002590287 |
| norm_vF4w | degen_AF | -0.720640074 | 0.000154895 | 0.003185018 |
| torque_range | axial_hyst | -0.719932242 | 0.000158344 | 0.003189507 |
| norm_vF6w | degen_border | -0.717428294 | 0.000171088 | 0.003377268 |
| norm_vF6w | degen_NPmatrix | -0.713848181 | 0.000190845 | 0.003693414 |
| fold_calca_L1 | fold_calca_T13 | 0.781862745 | 0.000209162 | 0.003970052 |
| norm_vF2w | degen_NPcells | -0.705180131 | 0.000247038 | 0.004600493 |
| weight_6w | weight_pre | 0.824175824 | 0.000288704 | 0.005276865 |
| norm_vF2w | degen_AF | -0.697607917 | 0.000307282 | 0.005514311 |
| degen_total | IVDheight_45 | -0.82081454 | 0.00032095 | 0.005656747 |
| fold_tac1_L4 | fold_tac1_L3 | 0.750257998 | 0.000335298 | 0.00580595 |
| norm_vF2w | degen_total | -0.693850487 | 0.000341606 | 0.005813184 |
| tors_stiff | S1 | 0.69282891 | 0.000351489 | 0.005879999 |
| torque_range | axialROM | -0.690570299 | 0.000374219 | 0.0061559 |
| norm_vF6w | IVDheight_56 | 0.813794685 | 0.000397687 | 0.006434705 |
| norm_vF4w | degen_total | -0.686137016 | 0.00042253 | 0.006726403 |
| norm_vF4w | degen_NPcells | -0.683672783 | 0.000451631 | 0.007075555 |
| degen_endplate | IVDheight_45 | -0.802759896 | 0.000547602 | 0.008445042 |
| degen_border | IVDheight_45 | -0.800319384 | 0.000586203 | 0.008901265 |
| norm_vF4w | IVDheight_percent | 0.795289153 | 0.000672663 | 0.010059373 |
| tors_stiff | axial_hyst | -0.664596273 | 0.000741137 | 0.010917948 |
| IVDheight_percent | IVDheight_34 | 0.789010989 | 0.000794614 | 0.011509776 |
| norm_vF4w | IVDheight_56 | 0.788530321 | 0.000804635 | 0.011509776 |
| injury | IVDheight_45 | NA | 0.000900968 | 0.012703646 |
| norm_vF4w | IVDheight_45 | 0.78177149 | 0.000956571 | 0.013297691 |
| S1 | axialROM | -0.653303219 | 0.000978019 | 0.013407014 |
| degen_NPcells | IVDheight_45 | -0.778170001 | 0.00104642 | 0.013956979 |
| degen_NPcells | IVDheight_percent | -0.778170001 | 0.00104642 | 0.013956979 |
| axialROM | tens_stiff | -0.649915302 | 0.001060584 | 0.013957291 |
| degen_NPmatrix | IVDheight_percent | -0.775616752 | 0.001114104 | 0.014338729 |
| degen_border | IVDheight_percent | -0.775450703 | 0.001118624 | 0.014338729 |
| norm_vF4w | degen_endplate | -0.64370933 | 0.001227275 | 0.015369368 |
| fold_tac1_L3 | fold_tac1_L2 | 0.715686275 | 0.001235509 | 0.015369368 |
| degen_NPmatrix | IVDheight_45 | -0.770986204 | 0.001245744 | 0.015369368 |
| torque_range | S1 | 0.642010164 | 0.001276619 | 0.015555844 |
| fold_calca_L2 | fold_calca_L1 | 0.696281363 | 0.001327887 | 0.015983219 |
| norm_vF4w | degen_border | -0.638867261 | 0.001372341 | 0.016319287 |
| IVDheight_percent | IVDheight_45 | 0.762637363 | 0.001514231 | 0.017711892 |
| norm_vF6w | IVDheight_percent | 0.761900531 | 0.001539974 | 0.017711892 |
| degen_AF | IVDheight_45 | -0.761806447 | 0.001543285 | 0.017711892 |
| norm_vF4w | degen_NPmatrix | -0.63286178 | 0.001572265 | 0.017837072 |
| degen_NPcells | IVDheight_56 | -0.755482829 | 0.001779226 | 0.019955633 |
| fold_calca_L4 | fold_calca_L3 | 0.682146543 | 0.001817081 | 0.020151224 |
| tors_stiff | tens_stiff | 0.623941276 | 0.001914513 | 0.020995824 |
| norm_vF2w | IVDheight_56 | 0.74572439 | 0.002198628 | 0.023846663 |
| norm_vF2w | degen_NPmatrix | -0.614788223 | 0.002329156 | 0.02498779 |
| fold_tac1_L5 | norm_vF2w | -0.639034937 | 0.002419725 | 0.025581362 |
| tors_hyst | axial_hyst | -0.612648221 | 0.00243632 | 0.025581362 |
| S2 | S1 | 0.607001694 | 0.002739216 | 0.028459014 |
| fold_tac1_L4 | fold_calca_L2 | 0.645614035 | 0.002830189 | 0.028937595 |
| norm_vF2w | degen_border | -0.605171976 | 0.002843918 | 0.028937595 |
| tors_hyst | torque_range | 0.603613778 | 0.002935716 | 0.029566854 |
| tors_stiff | creep_displacement | -0.600225861 | 0.00314393 | 0.031344033 |
| degen_NPmatrix | IVDheight_56 | -0.724680726 | 0.003369202 | 0.033254026 |
| degen_border | IVDheight_56 | -0.723452551 | 0.003450214 | 0.03371645 |
| degen_endplate | IVDheight_56 | -0.722483906 | 0.003515182 | 0.034014551 |
| fold_tac1_L5 | axialROM | 0.619548872 | 0.003575853 | 0.034265698 |
| norm_vF2w | IVDheight_percent | 0.711930233 | 0.004287263 | 0.040687774 |
| fold_calca_L5 | weight_pre | 0.600225691 | 0.005141753 | 0.048332482 |

**Supplemental Table S2. Correlation table for correlation pairs in female network analysis.**

| Variable 1 | Variable 2 | Rho | p | p.adj |
| --- | --- | --- | --- | --- |
| injury | norm_vF6w | NA | 3.68E-17 | 3.63E-14 |
| injury | norm_vF4w | NA | 7.60E-17 | 3.75E-14 |
| degen_NPmatrix | degen_border | 0.97748079 | 5.52E-15 | 1.81E-12 |
| injury | norm_vF2w | NA | 1.75E-14 | 4.31E-12 |
| degen_total | degen_NPcells | 0.958656461 | 2.22E-12 | 4.38E-10 |
| degen_total | degen_border | 0.942970445 | 5.18E-11 | 8.53E-09 |
| degen_total | degen_NPmatrix | 0.93327152 | 2.39E-10 | 3.38E-08 |
| torque_range | tors_stiff | 0.928853755 | 4.46E-10 | 5.50E-08 |
| degen_NPmatrix | degen_NPcells | 0.924623335 | 7.81E-10 | 8.56E-08 |
| degen_NPcells | degen_border | 0.922091226 | 1.08E-09 | 1.06E-07 |
| degen_total | degen_endplate | 0.90220285 | 9.60E-09 | 8.61E-07 |
| norm_vF4w | norm_vF2w | 0.897655424 | 1.48E-08 | 1.22E-06 |
| degen_total | degen_AF | 0.893850978 | 2.10E-08 | 1.60E-06 |
| Se | comp_stiff | 0.890708875 | 2.78E-08 | 1.96E-06 |
| norm_vF6w | norm_vF4w | 0.877903301 | 7.95E-08 | 5.05E-06 |
| degen_NPcells | degen_AF | 0.87753087 | 8.19E-08 | 5.05E-06 |
| injury | degen_endplate | NA | 3.15E-07 | 1.78E-05 |
| norm_vF6w | norm_vF2w | 0.857995742 | 3.30E-07 | 1.78E-05 |
| fold_tac1_L3 | fold_tac1_T13 | 0.911764706 | 3.51E-07 | 1.78E-05 |
| degen_endplate | degen_NPcells | 0.856702873 | 3.60E-07 | 1.78E-05 |
| creep_displacement | S2 | -0.855448899 | 3.90E-07 | 1.83E-05 |
| injury | degen_total | NA | 4.49E-07 | 2.01E-05 |
| creep_displacement | axialROM | 0.849802372 | 5.59E-07 | 2.40E-05 |
| injury | degen_AF | NA | 9.90E-07 | 4.07E-05 |
| degen_endplate | degen_AF | 0.833652224 | 1.45E-06 | 5.71E-05 |
| injury | degen_NPcells | NA | 1.51E-06 | 5.73E-05 |
| degen_border | degen_AF | 0.806938607 | 5.70E-06 | 0.000208186 |
| injury | IVDheight_percent | NA | 6.17E-06 | 0.000217601 |
| degen_endplate | degen_border | 0.802365994 | 7.05E-06 | 0.000240011 |
| degen_NPmatrix | degen_AF | 0.794356085 | 1.01E-05 | 0.000333047 |
| degen_endplate | degen_NPmatrix | 0.786922106 | 1.40E-05 | 0.000444644 |
| norm_vF6w | degen_NPcells | -0.777723632 | 2.04E-05 | 0.000630653 |
| creep_displacement | S1 | -0.765104461 | 3.35E-05 | 0.000974697 |
| norm_vF6w | degen_total | -0.764739052 | 3.40E-05 | 0.000974697 |
| norm_vF6w | degen_AF | -0.764231566 | 3.47E-05 | 0.000974697 |
| injury | degen_border | NA | 3.58E-05 | 0.000974697 |
| S2 | axialROM | -0.76284585 | 3.65E-05 | 0.000974697 |
| axial_hyst | weight_6w | 0.876923077 | 3.83E-05 | 0.000994714 |
| injury | degen_NPmatrix | NA | 4.97E-05 | 0.001257702 |
| S1 | axial_hyst | -0.751552795 | 5.53E-05 | 0.001363686 |
| tors_stiff | axialROM | -0.744776962 | 7.01E-05 | 0.001688085 |
| norm_vF2w | degen_endplate | -0.744024611 | 7.20E-05 | 0.001691281 |
| IVDheight_percent | IVDheight_56 | 0.859340659 | 8.20E-05 | 0.001882414 |
| axial_hyst | axialROM | 0.739130435 | 8.50E-05 | 0.001907732 |
| injury | IVDheight_56 | NA | 0.000102365 | 0.002245204 |
| norm_vF6w | degen_endplate | -0.731379137 | 0.000109985 | 0.002359897 |
| creep_displacement | axial_hyst | 0.72783738 | 0.000123347 | 0.002590287 |
| norm_vF4w | degen_AF | -0.720640074 | 0.000154895 | 0.003185018 |
| torque_range | axial_hyst | -0.719932242 | 0.000158344 | 0.003189507 |
| norm_vF6w | degen_border | -0.717428294 | 0.000171088 | 0.003377268 |
| norm_vF6w | degen_NPmatrix | -0.713848181 | 0.000190845 | 0.003693414 |
| fold_calca_L1 | fold_calca_T13 | 0.781862745 | 0.000209162 | 0.003970052 |
| norm_vF2w | degen_NPcells | -0.705180131 | 0.000247038 | 0.004600493 |
| weight_6w | weight_pre | 0.824175824 | 0.000288704 | 0.005276865 |
| norm_vF2w | degen_AF | -0.697607917 | 0.000307282 | 0.005514311 |
| degen_total | IVDheight_45 | -0.82081454 | 0.00032095 | 0.005656747 |
| fold_tac1_L4 | fold_tac1_L3 | 0.750257998 | 0.000335298 | 0.00580595 |
| norm_vF2w | degen_total | -0.693850487 | 0.000341606 | 0.005813184 |
| tors_stiff | S1 | 0.69282891 | 0.000351489 | 0.005879999 |
| torque_range | axialROM | -0.690570299 | 0.000374219 | 0.0061559 |
| norm_vF6w | IVDheight_56 | 0.813794685 | 0.000397687 | 0.006434705 |
| norm_vF4w | degen_total | -0.686137016 | 0.00042253 | 0.006726403 |
| norm_vF4w | degen_NPcells | -0.683672783 | 0.000451631 | 0.007075555 |
| degen_endplate | IVDheight_45 | -0.802759896 | 0.000547602 | 0.008445042 |
| degen_border | IVDheight_45 | -0.800319384 | 0.000586203 | 0.008901265 |
| norm_vF4w | IVDheight_percent | 0.795289153 | 0.000672663 | 0.010059373 |
| tors_stiff | axial_hyst | -0.664596273 | 0.000741137 | 0.010917948 |
| IVDheight_percent | IVDheight_34 | 0.789010989 | 0.000794614 | 0.011509776 |
| norm_vF4w | IVDheight_56 | 0.788530321 | 0.000804635 | 0.011509776 |
| injury | IVDheight_45 | NA | 0.000900968 | 0.012703646 |
| norm_vF4w | IVDheight_45 | 0.78177149 | 0.000956571 | 0.013297691 |
| S1 | axialROM | -0.653303219 | 0.000978019 | 0.013407014 |
| degen_NPcells | IVDheight_45 | -0.778170001 | 0.00104642 | 0.013956979 |
| degen_NPcells | IVDheight_percent | -0.778170001 | 0.00104642 | 0.013956979 |
| axialROM | tens_stiff | -0.649915302 | 0.001060584 | 0.013957291 |
| degen_NPmatrix | IVDheight_percent | -0.775616752 | 0.001114104 | 0.014338729 |
| degen_border | IVDheight_percent | -0.775450703 | 0.001118624 | 0.014338729 |
| norm_vF4w | degen_endplate | -0.64370933 | 0.001227275 | 0.015369368 |
| fold_tac1_L3 | fold_tac1_L2 | 0.715686275 | 0.001235509 | 0.015369368 |
| degen_NPmatrix | IVDheight_45 | -0.770986204 | 0.001245744 | 0.015369368 |
| torque_range | S1 | 0.642010164 | 0.001276619 | 0.015555844 |
| fold_calca_L2 | fold_calca_L1 | 0.696281363 | 0.001327887 | 0.015983219 |
| norm_vF4w | degen_border | -0.638867261 | 0.001372341 | 0.016319287 |
| IVDheight_percent | IVDheight_45 | 0.762637363 | 0.001514231 | 0.017711892 |
| norm_vF6w | IVDheight_percent | 0.761900531 | 0.001539974 | 0.017711892 |
| degen_AF | IVDheight_45 | -0.761806447 | 0.001543285 | 0.017711892 |
| norm_vF4w | degen_NPmatrix | -0.63286178 | 0.001572265 | 0.017837072 |
| degen_NPcells | IVDheight_56 | -0.755482829 | 0.001779226 | 0.019955633 |
| fold_calca_L4 | fold_calca_L3 | 0.682146543 | 0.001817081 | 0.020151224 |
| tors_stiff | tens_stiff | 0.623941276 | 0.001914513 | 0.020995824 |
| norm_vF2w | IVDheight_56 | 0.74572439 | 0.002198628 | 0.023846663 |
| norm_vF2w | degen_NPmatrix | -0.614788223 | 0.002329156 | 0.02498779 |
| fold_tac1_L5 | norm_vF2w | -0.639034937 | 0.002419725 | 0.025581362 |
| tors_hyst | axial_hyst | -0.612648221 | 0.00243632 | 0.025581362 |
| S2 | S1 | 0.607001694 | 0.002739216 | 0.028459014 |
| fold_tac1_L4 | fold_calca_L2 | 0.645614035 | 0.002830189 | 0.028937595 |
| norm_vF2w | degen_border | -0.605171976 | 0.002843918 | 0.028937595 |
| tors_hyst | torque_range | 0.603613778 | 0.002935716 | 0.029566854 |
| tors_stiff | creep_displacement | -0.600225861 | 0.00314393 | 0.031344033 |
| degen_NPmatrix | IVDheight_56 | -0.724680726 | 0.003369202 | 0.033254026 |
| degen_border | IVDheight_56 | -0.723452551 | 0.003450214 | 0.03371645 |
| degen_endplate | IVDheight_56 | -0.722483906 | 0.003515182 | 0.034014551 |
| fold_tac1_L5 | axialROM | 0.619548872 | 0.003575853 | 0.034265698 |
| norm_vF2w | IVDheight_percent | 0.711930233 | 0.004287263 | 0.040687774 |
| fold_calca_L5 | weight_pre | 0.600225691 | 0.005141753 | 0.048332482 |

**Supplemental Table S3. qPCR primer sequences.**

| Gene | Direction | Sequence |
| --- | --- | --- |
| Calca | Sense | 5’-CCACCTGCGTGACCCATCG-3’ |
|  | Antisense | 5’-TTCACCACACCTCCCGACC-3’ |
| Tac1 | Sense | 5’-GCTCTTTTGCCTATTAGTCC-3’ |
|  | Antisense | 5’-GCCCTTTGAGCATCTTCTTCA-3’ |
| Gapdh | Sense | 5’-AGCAACTCCCATTCTTCCAC-3’ |
|  | Antisense | 5’-TCAGTATCCTTGCTGGGCTG-3’ |

**Supplemental Table S4. Variables included in the trait correlation network analysis.**

| variable label | variable |
| --- | --- |
| sex | rat sex |
| injury | sham or injury group |
| testcage | number of cage tested in for von Frey |
| surgdate | date of surgery |
| weight_pre | pre-operative weight |
| weight_6w | weight at 6 weeks post-op |
| estrous | estrous stage at euthanization (females only) |
| IVDheight_pre_12 | pre-op height of L1/2 IVD |
| IVDheight_pre_23 | pre-op height of L2/3 IVD |
| IVDheight_pre_34 | pre-op height of L3/4IVD |
| IVDheight_pre_45 | pre-op height of L4/5 IVD |
| IVDheight_pre_56 | pre-op height of L5/6 IVD |
| IVDheight_pre_avg | average height of L3/4, L4/5, and L5/6 IVDs at pre-op |
| IVDheight_6w_12 | height of L1/2 IVD at 6 weeks |
| IVDheight_6w_23 | height of L2/3 IVD at 6 weeks |
| IVDheight_6w_34 | height of L3/4 IVD at 6 weeks |
| IVDheight_6w_45 | height of L4/5 IVD at 6 weeks |
| IVDheight_6w_56 | height of L5/6 IVD at 6 weeks |
| IVDheight_6w_avg | average height of L3/4, L4/5, and L5/6 IVD at 6 weeks |
| IVDheight_percent | percent change in average IVD height, pre-op to 6 weeks |
| tens_stiff | tensile stiffness |
| comp_stiff | compressive stiffness |
| axialROM | axial range of motion |
| axial_hyst | axial hysteresis |
| Se | creep elastic stiffness |
| S1 | creep fast response stiffness |
| S2 | creep slow response stiffness |
| tau1 | creep fast time constant |
| tau2 | creep slow time constant |
| creep_displacement | creep total displacement |
| tors_stiff | torsional stiffness |
| torque_range | torque range |
| tors_hyst | torsional hysteresis |
| degen_AF | degeneration grade – annulus fibrosus integrity |
| degen_border | degeneration grade – annulus fibrosus/nucleus pulposus border |
| degen_NPcells | degeneration grade – nucleus pulposus cellularity |
| degen_NPmatrix | degeneration grade – nucleus pulposus matrix quality |
| degen_endplate | degeneration grade – endplate quality |
| degen_total | total degeneration grade |
| vF_pre1 | first pre-op von Frey threshold |
| vF_pre2 | second pre-op von Frey threshold |
| vF2w | 2 week von Frey threshold |
| vF4w | 4 week von Frey threshold |
| vF6w | 6 week von Frey threshold |
| norm_vFpre | second pre-op von Frey threshold normalized to second pre-op von Frey threshold |
| norm_vF2w | 2 week von Frey threshold normalized to second pre-op von Frey threshold |
| norm_vF4w | 4 week von Frey threshold normalized to second pre-op von Frey threshold |
| norm_vF6w | 6 week von Frey threshold normalized to second pre-op von Frey threshold |
| ct_gapdh_T13 | Ct value for Gapdh gene in T13 DRG |
| ct_gapdh_L1 | Ct value for Gapdh gene in L1 DRG |
| ct_gapdh_L2 | Ct value for Gapdh gene in L2 DRG |
| ct_gapdh_L3 | Ct value for Gapdh gene in L3 DRG |
| ct_gapdh_L4 | Ct value for Gapdh gene in L4 DRG |
| ct_gapdh_L5 | Ct value for Gapdh gene in L5 DRG |
| ct_calca_T13 | Ct value for Calca gene in T13 DRG |
| ct_calca_L1 | Ct value for Calca gene in L1 DRG |
| ct_calca_L2 | Ct value for Calca gene in L2 DRG |
| ct_calca_L3 | Ct value for Calca gene in L3 DRG |
| ct_calca_L4 | Ct value for Calca gene in L4 DRG |
| ct_calca_L5 | Ct value for Calca gene in L5 DRG |
| ct_tac1_T13 | Ct value for Tac1 gene in T13 DRG |
| ct_tac1_L1 | Ct value for Tac1 gene in L1 DRG |
| ct_tac1_L2 | Ct value for Tac1 gene in L2 DRG |
| ct_tac1_L3 | Ct value for Tac1 gene in L3 DRG |
| ct_tac1_L4 | Ct value for Tac1 gene in L4 DRG |
| ct_tac1_L5 | Ct value for Tac1 gene in L5 DRG |
| fold_calca_T13 | fold change for Calca gene in T13 DRG |
| fold_calca_L1 | fold change for Calca gene in L1 DRG |
| fold_calca_L2 | fold change for Calca gene in L2 DRG |
| fold_calca_L3 | fold change for Calca gene in L3 DRG |
| fold_calca_L4 | fold change for Calca gene in L4 DRG |
| fold_calca_L5 | fold change for Calca gene in L5 DRG |
| fold_tac1_T13 | fold change for Tac1 gene in T13 DRG |
| fold_tac1_L1 | fold change for Tac1 gene in L1 DRG |
| fold_tac1_L2 | fold change for Tac1 gene in L2 DRG |
| fold_tac1_L3 | fold change for Tac1 gene in L3 DRG |
| fold_tac1_L4 | fold change for Tac1 gene in L4 DRG |
| fold_tac1_L5 | fold change for Tac1 gene in L5 DRG |
